# Correction to A Single‐Plasmid Inducible‐Replication System for High‐Yield Production of Short Ff (f1, M13 or fd)‐Phage‐Derived Nanorods

**DOI:** 10.1111/1751-7915.70192

**Published:** 2025-07-06

León‐Quezada, R. I., M. G. Miró, S. Khanum, A. J. Sutherland‐Smith, V. A. M. Gold, and J. Rakonjac. 2025. Microbial Biotechnology, 18: e70113. https://doi.org/10.1111/1751‐7915.70113

In Supplementary Information file *mbt270113‐sup‐0001‐supinfo.docx*, the image (graphics) of Fig. S6 on P7 is incorrect.

The correct image is:

**Figure:**
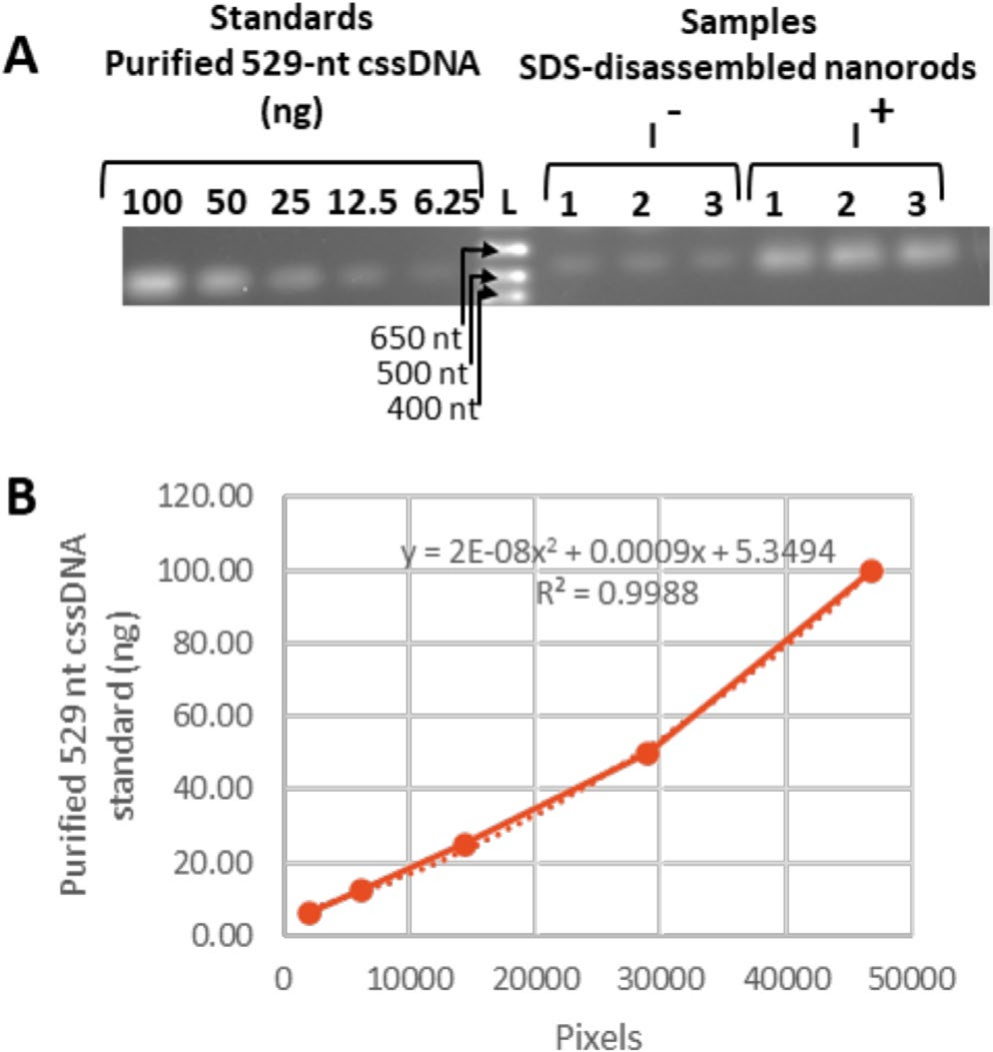
**FIGURE S6**. Quantification of the 529‐nt nanorods produced by the pPop‐up529LacUni plasmid.

We apologise for this error.

